# The Principles and Practice of Hydrotherapy

**Published:** 1899-02

**Authors:** 


THE PRINCIPLES AND PRACTICE OF HYDROTHERAPY. A Guide to the Application of Water in Disease. For Students and Practitioners of Medicine. By SIMON BARUCH, M. D., Visiting Physician to the J. Hood Wright (formerly Manhattan General) Hospital; Consulting Physician to the Montifiore Home for Chronic Invalids, etc., etc. With numerous illustrations. Octavo, pp. viii.—435. New York: William Wood & Co. 1898.

Dumoulin, the most celebrated physician of his time, as he lay upon his dying bed surrounded by his colleagues said: "Gentlemen—I leave behind me three most excellent physicians," each one present expected to be one of the chosen three, but to their consternation he named "water, exercise and regimen." These remedial agents have stood the test through all the ages and through all the "pathies." Modern medicine is tending toward monotherapy and less drug giving, to more direct, more positive and satisfactory methods. This trend is notable among educated physicians and craved by the laity. This tendency opens a fertile field for the browsing of the charlatan and the quack. Hence we have the mind curists and the faith curists, the christian scientists and the osteopathists.

Simon Baruch, a master of his subject, has written a volume or text-book, setting forth the curative value of water as a remedial agent. Dr. Baruch, in his preface says: "This book is written by a general practitioner for the guidance of his colleagues," thus differing from all other works, upon the subject. "Hydrotherapy" represents his work in all branches of medicine for a third of a century, and data accumulated from observations in over 100,000 cases.

It has been said that one who could use opium well was already a good physician, and it might be added that one who knew well the therapeutic value of water would add greatly to his fame and success. Every practising physician should read and study this most excellent and useful work—one that does not follow the beaten paths of "text-bookism." B. H. D.

